# Design and evaluation of the immunogenicity and efficacy of a biomimetic particulate formulation of viral antigens

**DOI:** 10.1038/s41598-017-13915-x

**Published:** 2017-10-23

**Authors:** Victor Riitho, Adam A. Walters, Satyanarayana Somavarapu, Benjamin Lamp, Till Rümenapf, Thomas Krey, Felix A. Rey, Ernesto Oviedo-Orta, Graham R. Stewart, Nicolas Locker, Falko Steinbach, Simon P. Graham

**Affiliations:** 1 Virology Department, Animal and Plant Health Agency, Woodham Lane, Addlestone, KT15 3NB United Kingdom; 2 Faculty of Health and Medical Sciences, University of Surrey, Guildford, GU2 7XH United Kingdom; 3 UCL School of Pharmacy, 29-39 Brunswick Square, London, WC1N 1AX United Kingdom; 4 Institute for Virology, University of Veterinary Medicine Vienna, Veterinaerplatz 1, 1210 Vienna, Austria; 5 Institut Pasteur, Unité de Virologie Structurale, Department Virologie, Paris CNRS UMR, 3569 Paris, France; 6 Hannover Medical School, Carl-Neuberg-Str. 1, 30625 Hannover, Germany; 7 German Center for Infection Research (DZIF), 30625 Hannover, Germany; 8 Present Address: The Pirbright Institute, Ash Road, Pirbright, Woking, GU24 0NF United Kingdom; 9 Present Address: International Livestock Research Institute, P.O. Box 30709, Nairobi, 00100 Kenya; 10 Present Address: The Jenner Institute, Old Road Campus Research Building, Roosevelt Drive, Oxford, OX3 7DQ United Kingdom; 11 Present Address: Sanofi Pasteur, 1541, Avenue Marcel Merieux – Campus Merieux, 69280 Marcy, L’Etoile France

## Abstract

Subunit viral vaccines are typically not as efficient as live attenuated or inactivated vaccines at inducing protective immune responses. This paper describes an alternative ‘biomimetic’ technology; whereby viral antigens were formulated around a polymeric shell in a rationally arranged fashion with a surface glycoprotein coated on to the surface and non-structural antigen and adjuvant encapsulated. We evaluated this model using BVDV E2 and NS3 proteins formulated in poly-(D, L-lactic-co-glycolic acid) (PLGA) nanoparticles adjuvanted with polyinosinic:polycytidylic acid (poly(I:C) as an adjuvant (Vaccine-NP). This Vaccine-NP was compared to ovalbumin and poly(I:C) formulated in a similar manner (Control-NP) and a commercial adjuvanted inactivated BVDV vaccine (IAV), all inoculated subcutaneously and boosted prior to BVDV-1 challenge. Significant virus-neutralizing activity, and E2 and NS3 specific antibodies were observed in both Vaccine-NP and IAV groups following the booster immunisation. IFN-γ responses were observed in *ex vivo* PBMC stimulated with E2 and NS3 proteins in both vaccinated groups. We observed that the protection afforded by the particulate vaccine was comparable to the licenced IAV formulation. In conclusion, the biomimetic particulates showed a promising immunogenicity and efficacy profile that may be improved by virtue of being a customisable mode of delivery.

## Introduction

Recently there has been a growing interest in using viral vectors or virus-like particles (VLPs) expressing heterologous antigens to induce T cell and B cell responses. Viral vectors are recombinant, attenuated viruses which express an antigen of interest following infection^1^, whereas, VLPs are self-assembling viral structural proteins tagged with antigen^2^. Viral vectors afford the opportunity to deliver antigen intracellularly, thus enabling direct access to the cytosolic MHC class I pathway^3^. Furthermore, they are inherently immunostimulatory, expressing pathogen-associated molecular patterns (PAMPs) that engage with pattern recognition receptors to enhance the induction of antigen-specific responses^4^. VLPs on the other hand display antigenic protein on the particle surface in a uniform and repeated fashion, which enhances activation of antigen-specific B cell receptors^5^. Together these approaches represent the next-generation of vaccines. However, this paper describes a third approach; ‘biomimetic’ particle vaccines, which have the advantages of both a viral vector and a VLP. In their simplest form biomimetic particles are polymeric particles, typically made of an inert polymer such as poly-(D, L-lactic-co-glycolic acid) (PLGA) or polycaprolactone (PCL), which encapsulate or are coated in antigen plus an immunostimulatory molecular adjuvant^6–8^. Biomimetic particles therefore provide a finely tuneable system, which allows for the spatial arrangement of multiple antigens whilst also accounting for nature of the immune response required through the incorporation of defined PAMPs.

We developed this approach by rationally designing a particle composed of a polymeric shell encapsulating a viral protein which is known to be a T cell target; often a non-structural protein only present during the intracellular viral replication cycle. Encapsulated protein has previously been shown to be efficiently cross-presented by MHC class I^9^. As well as antigen, a viral-associated nucleic acid PAMP, in this case polyinosinic-polycytidylic acid (poly(I:C)), is co-encapsulated as a surrogate for immune-stimulation provided by the replicating viral genome. Poly(I:C) is a synthetic double stranded RNA analogue that is an agonist for endosomal TLR-3 and the cytosolic receptors retinoic acid inducible gene I (RIG-I), melanoma differentiation-associated gene 5 (MDA-5) and DNA-dependent protein kinase catalytic subunits (DNA-PKcs)^10^. Poly(I:C) is a strong inducer of IL-12 and type I IFN thus enhancing dendritic cell (DC) maturation and polarizing antiviral T cell and antibody responses^11^. Co-delivery of PAMPs and antigen in biodegradable nanoparticles has been shown to enhance both T cell and humoral responses compared to inoculation of soluble antigen and TLR agonists^12,13^. In addition to the encapsulated payload, a second antigen, which is the major antibody target; often a viral envelope glycoprotein, is coated onto the surface of particles. Coating particles with antigen, as opposed to encapsulation of antigen, has shown to improve antibody responses by increasing antigen availability for engagement with B cell receptors^14^. The particles should fall roughly into the sub-micron size range, giving them access to the draining lymph nodes as well as an enhanced ability to be taken up by DCs^15^. DCs should be considered the key target in this approach, positioned at the interface of innate and adaptive system, they are the major professional antigen-presenting cells of the immune system, unparalleled in their ability to prime naïve T cells^16^. The shell of the particle here will be composed of PLGA, which is an innocuous polymer routinely used in drug delivery formulation which has been used pre-clinically as a vehicle for the delivery of antigens for human and veterinary applications^17,18^. Furthermore, PLGA particles have also been shown to be inherently immunogenic, activating the inflammasome and therefore driving the innate immune responses required for the induction of adaptive immunity^19^. Previous descriptions of biomimetic particles have focussed on the encapsulation of single model antigens with or without PAMPs, this study expands upon this concept, adding a second antigen spatially arranged so as to induce the relevant immune response i.e. a surface coated B cell antigen to readily enable BcR engagement and an encapsulated T cell antigen to prolong and augment antigen-presentation to TcR.

We chose to evaluate this approach using bovine virus diarrhoea virus (BVDV) as an example of a virus for which there is a need for improved vaccines. BVDV is an economically important pathogen that is endemic in cattle around the world. Infection by BVDV has a profound impact on the cattle industry as a result of production and reproductive losses^20^. Currently both live attenuated and inactivated vaccines are used to control BVDV^21^. Live attenuated vaccines, although efficacious, have shortcomings such as the requirement for cold chain storage and transportation and a limited shelf life, safety concerns in pregnant cattle, immunosuppression and possible reversion to virulence by mutation or genetic recombination with wild-type viruses^22^. Inactivated vaccines are inherently safer but require frequent booster immunisations and often fail to provide foetal protection. Inactivated vaccines require administration with a strong adjuvant, which may carry risks, as exemplified by the PregSure^®^ BVD vaccine which induced maternal alloantibodies linked to the fatal neonate-maternal incompatibility syndrome, bovine neonatal pancytopenia^23,24^. It has been proposed that inactivated BVDV vaccines lack efficacy because of their failure to induce potent T cell responses possibly due to absence of non-structural proteins, which have been shown to be targets of the T cell responses of immune cattle^25,26^. Attempts to develop subunit vaccines have focused on the structural envelope glycoprotein E2, which is the dominant target of neutralising antibodies. A variety of approaches have been experimentally evaluated in cattle, including plasmid DNA, eukaryotically expressed recombinant antigen, individually or combined using heterologous DNA prime-protein boost regimes or via live viral vectors^27–29^. Assessment of the specificity of the T cell response to BVDV has been limited to a number of viral proteins (core, E^rns^, E2, NS2 and NS3) that have been shown to be recognised by T cells^26,30^. Since NS3 is highly conserved it has been proposed that the inclusion of NS3 protein may increase the efficacy of E2 subunit vaccines^31^. We therefore formulated a biomimetic BVDV particle containing NS3 protein, which this study confirmed by proteome-wide peptide library screening to be an immunodominant T cell antigen, alongside poly(I:C), and with the E2 glycoprotein coated on to the surface as the neutralising antibody target. The use of BVDV as the model virus allowed the testing of the vaccine for both immunogenicity and efficacy in the large mammalian definitive host species, something that is seldom achieved in bioengineering projects. In this model, we show that biomimetic particles induce antibody and T cell responses in cattle that afford significant protection against persistent infection following high dose heterologous viral challenge.

## Results

### Design and characterisation of biomimetic particle vaccine formulations

**Figure 1:**
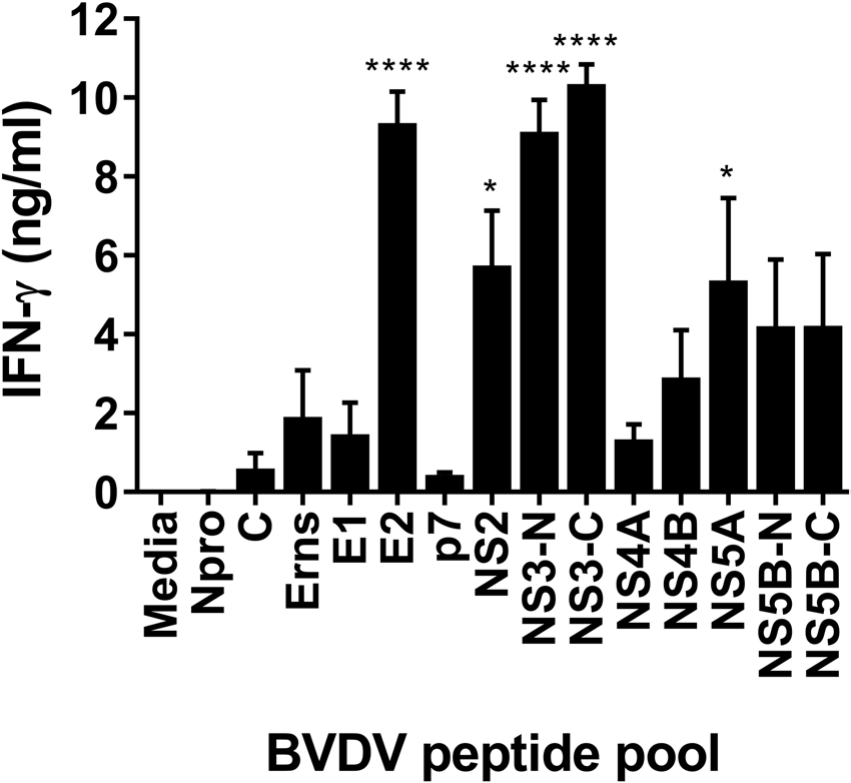
Recognition of BVDV-1 proteins by T cells from immune cattle. PBMC from BVDV-immune cattle challenged by experimental infection with BVDV Oregon C24v (n = 5) were isolated on day 21 post-challenge, and stimulated *in vitro* with synthetic peptides pooled to represent BVDV-1 proteins. PBMC cultured in media alone were included as a negative control. IFN-γ secreted into culture supernatants was quantified by ELISA after 48 hours. Mean data are presented and error bars show the SEM. Values for virus and peptide pool-stimulated conditions were compared to the unstimulated (media) control using a one-way ANOVA followed by a Dunnett’s multiple comparison test; ****p < 0.0001, and *p < 0.05.

**Figure 2:**
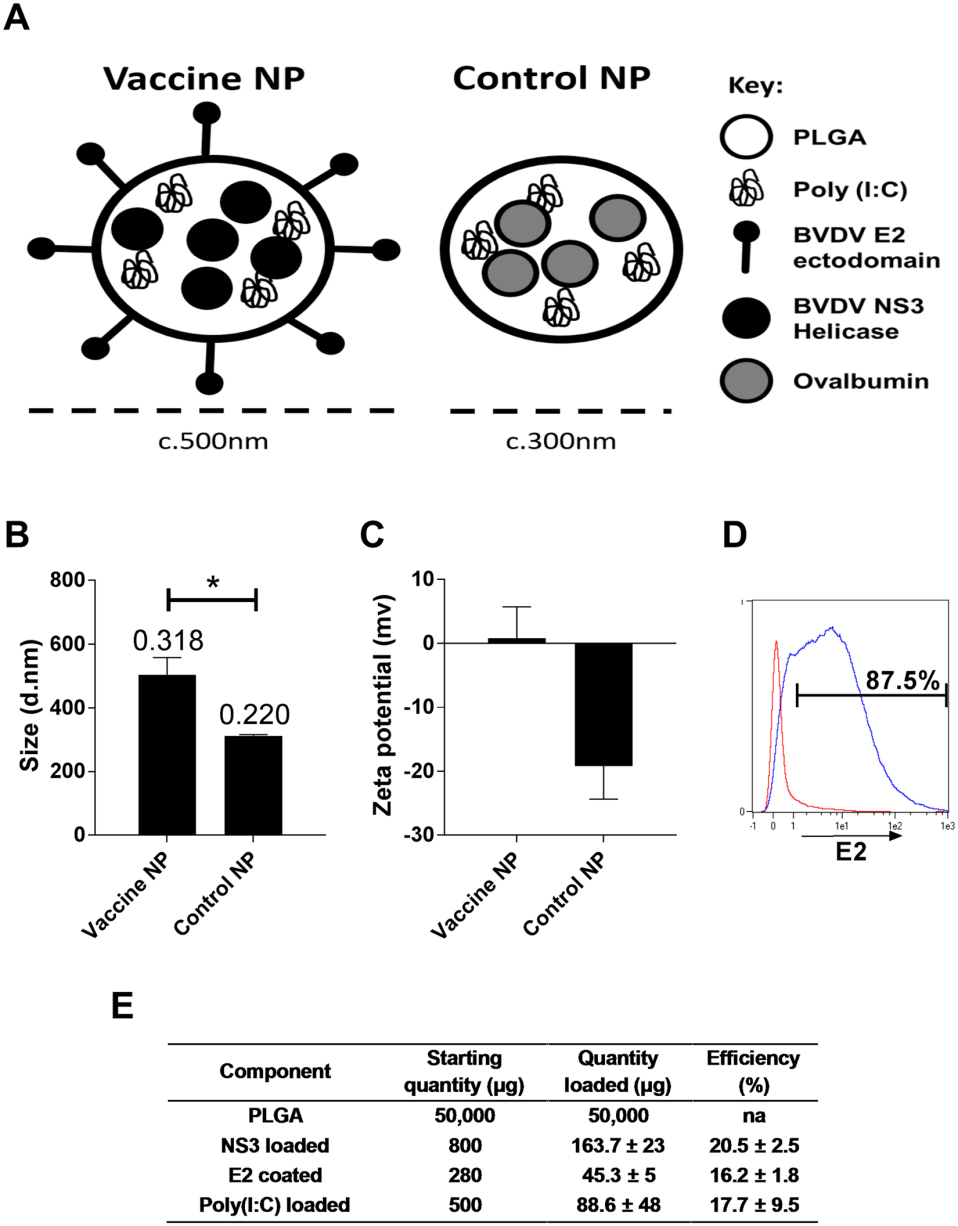
*In vitro* characterisation of nanoparticulate formulations of BVDV antigens. PLGA nanoparticles were synthesised as described with either BVDV antigens (Vaccine-NP) or OVA (Control-NP). A graphical representation of Vaccine-NP is shown in (**A**). Particles were analysed with dynamic light scattering to assess size (**B**) and charge (**C**); data represents the pooled particles used in this study read in triplicate runs. Size bars are annotated with polydispersity indices; error bars represent the SD. The size and charge of Vaccine-NP and Control-NP were compared by a student’s t-test (*p < 0.05). Surface coating of nanoparticles with BVDV E2 was determined by flow cytometry following staining of Control-NP (red) and Vaccine-NP (blue) with a BVDV E2 specific mAb followed by anti-mouse IgG1-APC (**D**). Calculation of nanoparticle loading and coating on a per dose basis and estimate of loading/coating efficiency (**E**). Mean data of all particle batches used in this study (n = 12) are presented ± SD.

T cell responses of immune cattle to BVDV peptide pools were assayed by IFN-γ ELISA 21 days following BVDV Oregon C24V challenge infection (Fig. 1). Significant IFN-γ responses were observed against the peptide pools representing the E2, NS2, NS3 and NS5A proteins. Since the greatest responses were observed to the E2 peptide pool and the NS3-C peptide pool, which represents the C-terminal helicase domain of NS3, the NS3-helicase domain was selected for encapsulation within the particle while E2, was chosen to be coated on to the surface of the biomimetic particles (Fig. 2A). Both the BVDV antigen loaded particles (Vaccine-NP) and OVA loaded particles (Control-NP) used in this study were characterised *in vitro* (Fig. 2). The Vaccine-NPs were 200 nm larger than the Control-NP; with particles being sized at ≈500 nm and ≈300 nm, respectively (Fig. 2B). The Vaccine-NP also exhibited a higher polydispersity index value of 0.318 compared to the Control-NP, which were slightly more uniform having a polydispersity index of 0.22. With regards to surface charge, after E2 coating, the vaccine particles increased their charge becoming effectively neutrally charged whereas the control particles remained negatively charged, possibly due to the capping of the terminal carboxyl group with tris (Fig. 2C). The surface E2 coating was also confirmed using flow cytometry (Fig. 2D). After being stained with a neutralising anti-E2 mAb, the fluorescent labelling of the vaccine particles far exceeded that of the control particles with nearly 90% of the particles staining positive for E2 suggesting both an efficient coating and preservation and availability of the neutralising E2 epitope on the particle surface. Vaccine-NP showed an NS3 and poly(I:C) loading efficiencies of 20.5% and 17.7%, respectively, and an E2 coating efficiency of 16.2% (Fig. 2E). This translated to 163 µg NS3, 45 μg E2 and 88 μg poly(I:C) in approximately 50 mg of PLGA particles per dose. Control-NP were loaded with poly(I:C) with a broadly similar efficiency and ovalbumin loaded at 20% (Data not shown). The poly(I:C) loading was variable between batches possibly because of the inherent solubility of poly(I:C) or alternately because the quantification relied on detecting the absence of poly(I:C) in wash supernatants rather than detecting the quantity loading.

### Antigen-specific antibody and T cell IFN-γ responses following vaccination with biomimetic formulation

**Figure 3:**
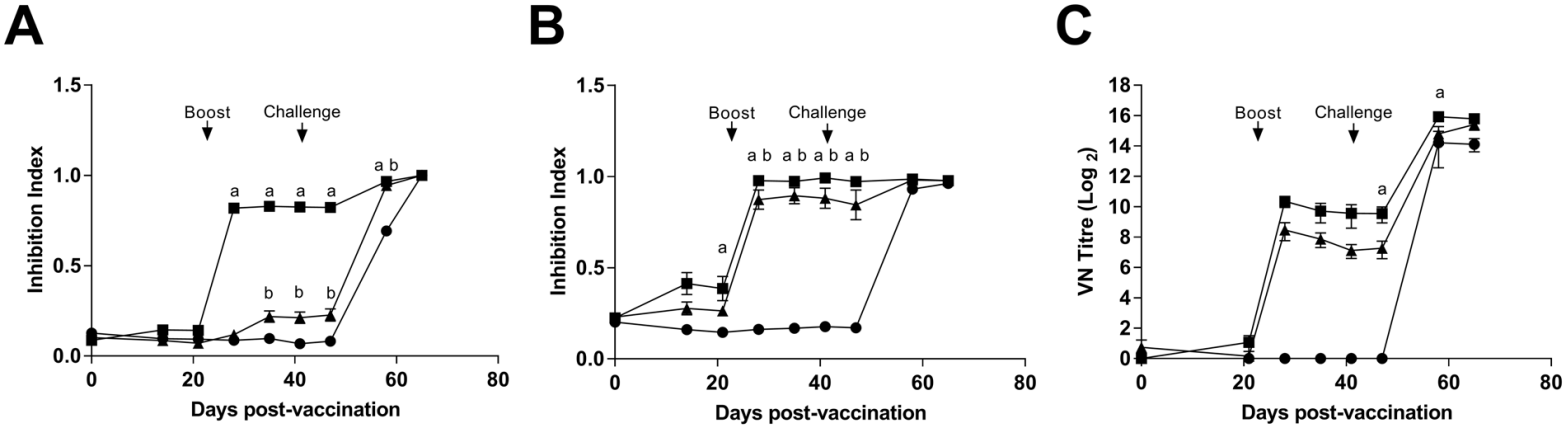
BVDV antigen-specific antibody responses following vaccination with nanoparticulate formulation of BVDV antigens and challenge infection. Sera from calves vaccinated with either inactivated BVDV vaccine (IAV) (squares), Vaccine-NP (triangles) or Control-NP (circles) were collected longitudinally over the course of vaccination and BVDV challenge. NS3 (**A**) and E2 (**B**) specific antibodies were measured by competitive ELISA and BVDV-neutralising titres determined by virus-neutralization assay (**C**). Mean data for each group (n = 6) are presented, error bars represent SEM and statistical analysis was performed using a two-way ANOVA followed by a Tukey’s multiple comparison test; statistically significant differences between the Control-NP group and the IAV and Vaccine-NP groups are indicated as ‘a’ and ‘b’, respectively. Booster immunisation and BVDV challenge infections on days 21 and 41 post-vaccination are indicated.

Serum antibody responses following vaccination and challenge were assessed by competition antibody ELISA against E2 and NS3 proteins and by determining virus neutralization titres (VNT) (Fig. 3). NS3 and E2 specific antibody levels were determined as percent inhibition indices (Fig. 3A,B). In the IAV group, NS3-specific antibody values were significantly greater than the Control-NP group from day 7 post-boost (28 days post-vaccination; dpv) until 58 dpv. The NS3 antibody response in the Vaccine-NP group showed a similar kinetic but with a lesser magnitude, being significantly greater than the Control-NP group from 35–58 dpv. Following IAV vaccination, significant E2 antibody values were detected on 21 dpv these were boosted by the second immunisation and remained significantly greater than those of the Control-NP group until 58 dpv (day 17 post-challenge). The E2-specific response of the Vaccine-NP immunised group showed a similar trend to the IAV group with significant responses compared to the control group 35–58 dpv. The neutralising antibody response, as expected, mirrored that measured by E2 ELISA. However, inter-animal variance in VNTs meant that only the IAV group on days 47 and 58 dpv (days 6 and 17 post-challenge) had titres significantly greater than the Control-NP group. Anti-ovalbumin antibody responses could be detected in the Control-NP group but not the Vaccine-NP or IAV groups (data not shown).

**Figure 4:**
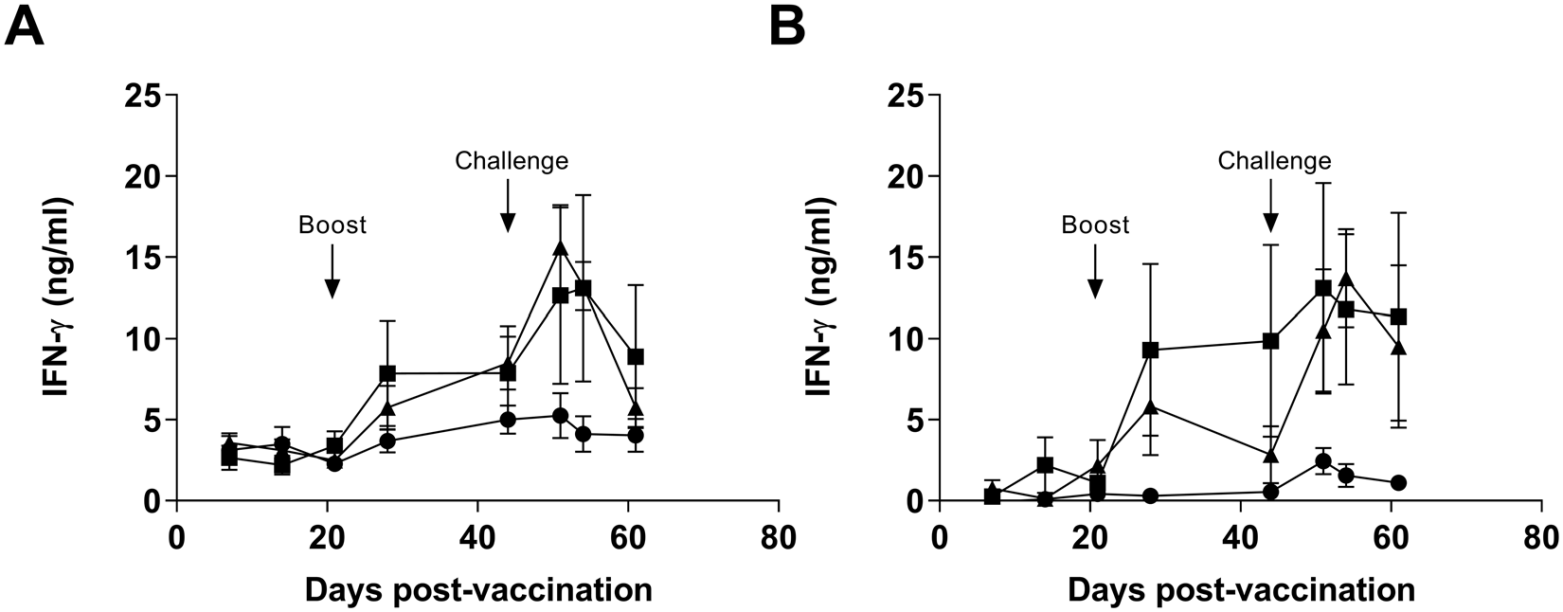
BVDV antigen-specific T cell IFN-γ responses following vaccination with nanoparticulate formulation of BVDV antigens and challenge infection. PBMCs from calves vaccinated with either inactivated BVDV vaccine (IAV) (squares), Vaccine-NP (triangles) or Control-NP (circles) were collected longitudinally over the course of vaccination and BVDV challenge, stimulated with recombinant BVDV E2 (**A**) or NS3 helicase (**B**) proteins. After 48 hours, IFN-γ in culture supernatants was measured by ELISA. Mean unstimulated-corrected data for each group (n = 6) are presented, error bars represent SEM. Booster immunisation and BVDV challenge infections on days 21 and 41 post-vaccination are indicated.

T cell responses were assessed by measuring IFN-γ levels following *ex vivo* stimulation of PBMC with E2 and NS3 proteins (Fig. 4). In both IAV and Vaccine-NP groups, IFN-γ responses to stimulation with both proteins were detected post-booster immunisation and further increased upon challenge. However, substantial variation in the magnitude of responses of individual animals in both groups meant that these responses did not achieve statistical significance.

### Assessment of vaccine-induced protection against BVDV challenge infection

**Figure 5:**
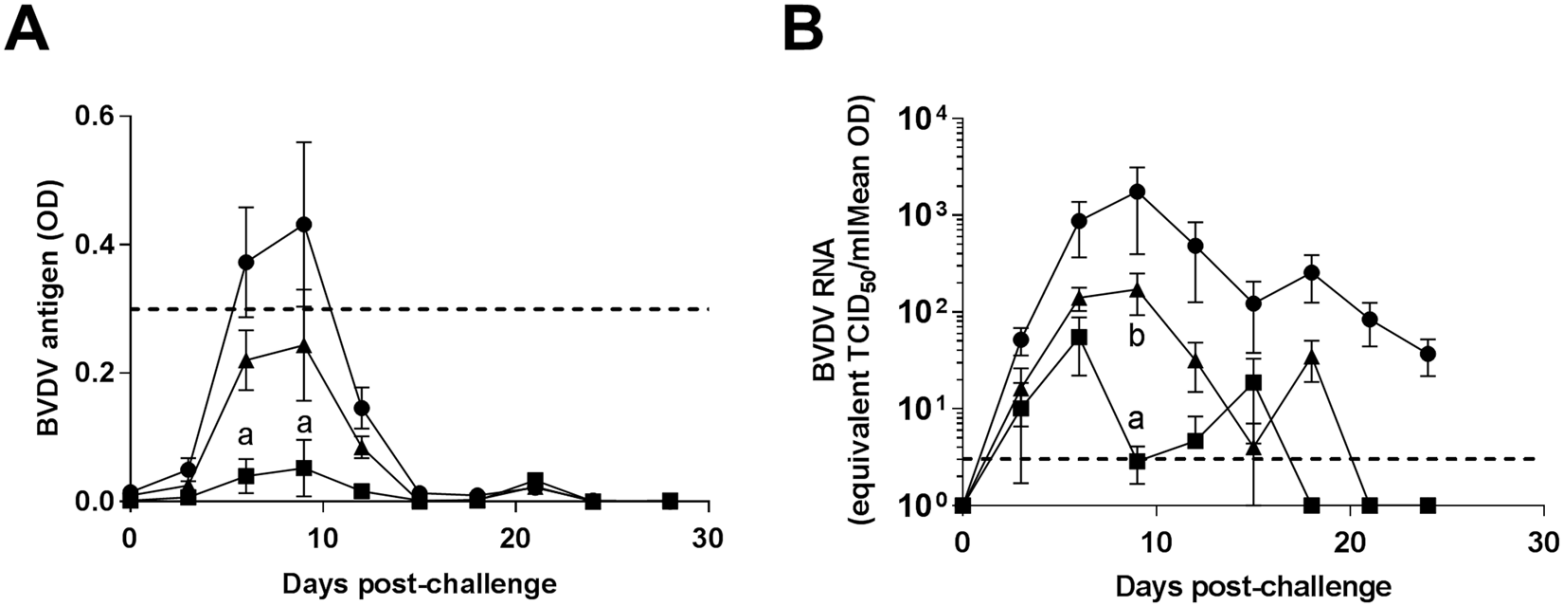
Detection of BVDV in the blood of vaccinated animals following challenge infection. Following BVDV-1 challenge infection, blood samples from inactivated BVDV vaccine (IAV) (squares), Vaccine-NP (triangles) or Control-NP (circles) vaccinated calves were assessed for BVDV E^rns^ antigen by ELISA (**A**) and BVDV RNA by quantitative RT-PCR (**B**). Mean data for each group (n = 6) are presented, error bars represent SEM and statistical analysis was performed using a two-way ANOVA followed by a Tukey’s multiple comparison test; statistically significant differences between Control-NP group and the IAV and Vaccine-NP groups are indicated as ‘a’ and ‘b’, respectively. Horizontal dashed lines show the assay cut-off for antigen positivity (COD >0.3) and detection limit (3.55 TCID_50_) for ELISA and RT-PCR assays, respectively.

Protection against BVDV challenge infection was assessed by measurement of viral antigen (E^rns^) and viral RNA in peripheral blood. As shown in Fig. 5, blood antigen levels became detectable at day 6 post-challenge and reached their peak at day 9 after which they began to decline. This trend was consistent between all three groups, however, there were differences between each group; the IAV group had the least blood antigen (statistically significant on day 6 and 9 post-challenge), the Vaccine-NP group had an intermediate level whilst the Control-NP group had the highest. The levels of BVDV RNA in blood followed a similar trend to that observed for viral antigen. They peaked at day 9 post-challenge in the case of the Vaccine and Control-NP groups; however, there were significantly higher levels of BVDV RNA in the Control-NP group compared to both Vaccine-NP and IAV groups at this peak. The IAV group showed a peak in viral RNA on day 6-post challenge. Interestingly, all groups showed a second peak in viral RNA, which was particularly apparent in the Vaccine-NP and IAV group, where the two groups show the same trend albeit offset. Viral RNA was cleared from the blood of the Vaccine-NP and IAV groups on day 21 and 18, respectively, whereas, viral RNA was still detectable in the blood of Control-NP animals at the termination of the experiment (day 24 post-challenge), which is in line with another observation the more pathogenic strains such as Ho916 induce a persistent infection in post-natal infection^32^.

## Discussion

Subunit vaccines based on a single protein have been used successfully to protect against a number of diseases. However, for diseases against which there is no vaccine or traditional vaccine approaches are suboptimal, it is likely that more rationally designed formulations are required. These formulations should take into account the physical nature of the antigen and how it interacts with the immune system. Since the licensure of GSK’s RTS,S, a malarial protein construct expressed on the surface of the hepatitis B viral capsid as a VLP, there has been a growing interest in using particulates as antigen carriers to induce more potent immune responses^33^. However the antigen need not be expressed as a fusion protein, it may simply be chemically linked to VLPs post-production. This strategy greatly enhances the immunogenicity when compared to free protein in a number of models^34,35^. The present study took an alternate approach using polymeric nanoparticles as the antigen carrier, in combination with recombinant BVDV antigens, E2 and NS3, and the molecular adjuvant poly(I:C) and compared this formulation to a commercial inactivated vaccine. While production of VLPs requires expensive industrial scale fermenters to produce sufficient quantities of inoculum, polymers such as PLGA particles can be produced using inexpensive, relatively simple methods and antigen can be conjugated post-synthesis. Furthermore, they may allow for a more readily customisable approach such as the inclusion of specific adjuvants. A rational approach was taken to the design of the vaccine particles incorporating multiple antigens. With regards to BVDV antigens, we have identified and utilised the two immunodominant T cell and B cell target antigens, NS3 helicase and E2 (Fig. 1 and^26,36–38^); other antigens could be included in future studies such as the NS3 N-terminal protease domain or the full-length NS3 protein, however, this is particularly hard to express as a recombinant protein. BVDV E2 protein was selected based on its ability to induce neutralising antibodies^39,40^. This study also confirmed NS3 as a second T cell antigen for inclusion in the formulation. PLGA was chosen as the particle shell due to its safety profile and ease of handling. Poly(I:C) was selected as the molecular adjuvant due its close association with viral infection, we speculated it may be able to induce an antiviral Th1/cytotoxic skewed immune response, similar to that of a virus, furthermore BVDV has been shown to modify TLR3 expression potentially as an immunological escape mechanism^41–43^. We observed a relatively low loading of antigen, however, this was in keeping with our previous results^44^, which we speculate is due to our selected process parameters. Indeed, other studies have reported a range of antigen loading efficiencies^8,45,46^. Future work should consist of improving the method to enable better loadings. The Vaccine-NP were slightly larger than the Control-NP, we believe this represents, firstly, physical differences between the encapsulated antigens and, secondly, the additional coated antigen whose structure has not been determined in this setting. In this study, we were unable to compare the vaccine formulations with unencapsulated antigens and adjuvant due to practical limitations, but the antigens we selected have previously been tested as protein vaccines with improved efficacy when combined^47^. Given the large amount of antigen used, a dose-response study will be necessary to determine the optimal amount of antigen to use.

In terms of magnitude of immune responses the Vaccine-NP did not perform as well as the commercial inactivated vaccine. This is likely due to the inclusion of the powerful adjuvant Quil-A in the inactivated formulation which cannot be used in humans due to adverse reactions induced^48^. In contrast, most studies generally compare experimental vaccines to a unadjuvanted formulation or a formulation containing Alum, which is not as potent and polarises towards Th2 responses. To elucidate the contribution of the biomimetic formulation to the immunogenicity a more informative comparison would be to compare the soluble form of the antigens to the spatially arranged rationally designed particle. However, in terms of translational potential we felt comparing the biomimetic particle to the commercial product would be more relevant. Responses may be enhanced, with the use of better adjuvants or immuno-stimulating polymers or combinations of such which stimulate broader immune responses. Also, combinations of TLR agonists may act synergistically in triggering the desired T cell or humoral immune responses^49^. A recent study, demonstrated enhanced antibody responses to synthetic nanoparticles containing antigen and TLR-4 and TLR-7 ligands compared to nanoparticles containing antigen and individual TLR ligands^12^. There is no reason why Quil-A or a safer derivative such as QS-21^50^ could not be included in a biomimetic nanoparticle or even in the more conventional, unencapsulated form alongside the particle, as has been done with alum^51^. The molecular adjuvant poly(I:C) used in the reported biomimetic formulation is currently being extensively studied and it is it highly plausible that poly(I:C) will be subsequently approved for both human and veterinary use^52,53^.

Biomimetic vaccine formulations offer a promising mode of vaccination that can be improved by use of better adjuvants, greater consideration of the location of antigen (coated or encapsulated), as well as targeted delivery of antigen and adjuvant to dendritic cells. In addition to the adaptability of this method of antigen delivery, other advantages such as the relative safety and capacity for differentiating infected from vaccinated animals (DIVA) are offered by the use of subunit antigens. Whether chemical conjugation of antigen to PLGA can match the immunogenicity warranted by antigen conjugated to VLPs in a similar fashion has yet to be demonstrated conclusively, nonetheless the prospect is exciting. We believe that biomimetic nanoparticulate formulations could be developed as both veterinary and humans vaccines, although the regulatory requirements will differ. In recent years, a number of studies have been published which have evaluated nanoparticulate delivery of vaccines for a number of major livestock pathogens in both cattle (foot-and-mouth disease virus; bovine parainfluenza-3 virus; bovine respiratory syncytial virus^54–57^) and pigs (swine influenza A virus; porcine reproductive and respiratory syndrome virus-1 and −2^58–60^). The selection of BVDV as the ‘model’ virus allowed us to test the biomimetic vaccine for both immunogenicity and efficacy in the large mammalian definitive host species, something that is seldom achieved in bioengineering projects. Since large animal models have been shown to more accurately predict vaccine outcome in humans than do other models^61^, our model may provide useful insights to guide human vaccine development.

## Materials and Methods

### Proteins and Peptides

The helicase domain of NS3 protein (BVDV polyprotein amino acids 1782–2272) was cloned from BVDV-1a strain CP7 (GenBank accession no. U63479.1^62^) and respective plasmid pL200 was expressed as a C-terminal fusion protein with a 12 His-tag in *Escherichia* coli Rosetta^TM^ cells (Novagen) and purified via HisTrap HP columns (GE Life Science, Freiburg, Germany) using native buffer (300 mM NaCl, 150 mM NaH_2_PO_4_, pH 7.4) conditions. Protein was eluted with gradients of 100–500 mM imidazole dissolved in the same buffer. Protein purity was assessed by SDS-PAGE and shown to be >90%. The ectodomain of E2 protein from BVDV-1a NCP7 and NADL (polyprotein amino acids 1782–2272) was expressed in insect cells. Briefly, the corresponding genes were cloned into a modified Drosophila S2 expression vector and transfection was performed as described before^63^. For large-scale production, cells were induced with 4 μM CdCl_2_ at a density of approximately 7 × 10^6^ cells/ml for 7 days, pelleted, and the soluble ectodomain was purified by affinity chromatography from the supernatant using a StrepTactin Superflow column followed by size exclusion chromatography using a Superdex200 column in 10 mM TRIS pH8, 150 mM NaCl. A library of overlapping 16mer peptides, offset by 4 amino acids spanning the entire length of the polyprotein of the BVDV-1 strain, Oregon C24V (GenBank accession no. AF091605), was synthesised by Pepscan Systems B.V. (Lelystad, The Netherlands) and peptides pooled to represent the BVDV proteins as described previously^64^.

### Viruses

The prototype BVDV-1a Oregon C24V strain and a BVDV-1a clinical field isolate Ho916^65^ were propagated *in vitro* by inoculation of foetal bovine turbinate (BT) cell monolayers (Cell and Tissue Culture Unit, APHA, Addlestone, UK). BT cells were maintained in Minimum Essential Medium (MEM) with 7.5% sodium carbonate, 5% lactalbumin hydrolysate, 1 mM sodium pyruvate, 2 mM glutamine, 100 U/ml penicillin, 100 μg/ml streptomycin and 20 μg/ml nystatin (all Invitrogen, Life Technologies, Paisley, UK) and 10% BVDV-free foetal bovine serum (FBS; Autogen Bioclear, Calne, UK) (BT medium). After 4 days, the supernatant was collected and pooled with a freeze-thawed cell lysate. The resultant pool was clarified by centrifugation at 524 × *g* for 10 minutes, aliquoted and stored at −80 °C. Virus titres were determined as the median (50%) tissue culture infectious dose (TCID_50_) using the Spearman-Kärber method^66^ following immunoperoxidase staining^67^. Mock virus supernatant was prepared in an identical manner from uninfected BT cells.

### Peptide epitope screening

All animal work was approved by the Animal and Plant Health Agency’s Institutional Animal Care and Use Committee and all procedures were conducted in accordance with the UK Animals (Scientific Procedures) Act 1986. To screen for BVDV T cell antigens, five 3–4 month old Holstein-Friesian calves were inoculated intranasally (i.n.) with 10^7^ TCID_50_ of BVDV Oregon C24V (2 ml divided equally between each nostril and administered using a mucosal atomization device MAD-300, Wolfe Tory Medical, USA). Animals were monitored for clinical signs of infection, viraemia and heparinised blood samples collected to assess T cell responses to BVDV peptide pools.

### Vaccine formulations

A formulation of PLGA (Resomer RG502 H, 50:50 LA:GA molar ratio, Mw 7–17 KDa, Sigma-Aldrich, Poole, UK) nanoparticles encapsulating BVDV-1 CP7 NS3 helicase domain and poly(I:C) (Sigma-Aldrich, Poole, UK) and coated with BVDV-1 NCP7 and NADL baculovirus-derived E2 protein ectodomain (Vaccine-NP; approximately 150 µg NS3, 50 µg E2 and 80 μg poly(I:C) per dose) was prepared as previously described^44^. Particles were synthesised sequentially; firstly, NS3 loaded particles were made, NS3 loading was then determined by hydrolysis of a representative fraction of particles with 0.1 M NaOH, and following neutralisation with 0.1 M HCl the protein content was determined using BCA assay (Thermo Fisher Scientific). E2 was then coated using a previously described method^44^ and coated protein was determined by subtracting protein content post-coating from the protein content prior to coating. E2 coating of particles was additionally assessed by staining particles with an E2-specific monoclonal antibody (clone WB214, APHA) and flow cytometric analysis using a MACSQuant Analyzer flow cytometer (Miltenyi Biotec, Bisley, UK)^44^. The poly(I:C) loading was calculated by subtracting the quantity of poly(I:C) detected in particle wash supernatant, as determined by absorbance OD_240_, from the starting quantity. PLGA nanoparticles similarly formulated with poly(I:C) and ovalbumin protein (Sigma-Aldrich) were used as a negative control (Control-NP). Particle size and surface charge were assessed by hydrodynamic diameter and zeta potential measurements using a Zetasizer 3000 (Malvern Instruments, Malvern, UK). A licensed inactivated BVDV vaccine (IAV), Bovidec^®^ (Novartis Animal Health, Camberley, United Kingdom), was used as a reference standard for both immunogenicity and efficacy. According to the manufacturer’s specifications, Bovidec consists of an inactivated BVDV-1a strain, KY1203nc^68^ in MEM (5 × 10^6^ TCID_50_ per 4 ml dose), which is adjuvanted with Quil-A (1 mg per dose).

### Vaccination and challenge of cattle

To evaluate vaccines, eighteen 6–8 month old male Holstein-Friesian calves were pre-tested for the absence of BVDV antibody and antigen and randomly assigned into three experimental groups prior to vaccination. Group sizes of 6 were calculated, using data from a previous published BVDV E2 vaccine trial^40^, to be able to detect significant differences in neutralising antibody titres between groups with 92% power and 95% confidence. Calves (n = 6) received a primary subcutaneous inoculation of Vaccine-NP or Control-NP in 2 ml or IAV in 4 ml. A similar booster inoculation was given at 21 days post-vaccination (dpv). On 41 dpv, all cattle received an intranasal inoculation with 5 × 10^6^ TCID_50_ BVDV-1a Ho916 in 2 ml BT medium, which was confirmed by back titration. Clinical scores and rectal temperatures were monitored prior to viral challenge and on a daily basis subsequently^32^. Blood samples were collected longitudinally over the course of vaccination and challenge to assess serum antibody and peripheral blood T cell IFN-γ responses, and to monitor for viral loads.

### Detection of antigen-specific and virus-neutralising antibody responses

Competitive ELISA kits were used for the detection of antibodies against NS3 (Pourquier^®^ ELISA BVD p80, Institut Pourquier, Montpellier, France) and E2 (VDPro^®^ BVDV, Jeno Biotech Inc., Chuncheon, Republic of Korea) according to the manufacturer’s instructions. To determine virus neutralising antibody titres, test sera were heat inactivated for 30 minutes at 56 °C after which a two-fold dilution in BT medium was added to duplicate wells of a flat-bottomed 96-well plate. Positive and negative control sera were also tested. An equal volume containing 100 TCID_50_ of BVDV-1 Ho916 was added and incubated for 1 hour at 37 °C. BT cells were then added (1.5 × 10^4^) and incubated at 37 °C for 5 days, after which immunoperoxidase staining for virus was conducted as described previously^67^. Neutralising titres were calculated as reciprocal of the highest serum dilution that inhibited virus growth by 50% using the Spearman–Kärber method.

### Detection antigen specific T cell IFN-γ responses

Peripheral blood mononuclear cells (PBMC) isolated by density centrifugation using Histopaque 1.077 (Sigma-Aldrich) were suspended at a density of 5 × 10^6^ cells/ml in RPMI-1640 medium supplemented with 10% FBS, 2 mM L-glutamine, 20 mM HEPES, 100U/ml penicillin and 100 μg/mL streptomycin, and 100 μl transferred to wells of 96 well round-bottom plates. Cells were stimulated in duplicate wells with 100 μl of peptide pools with each peptide at 2 μg/ml or recombinant E2 and NS3 proteins at 10 μg/ml. Medium alone was used as negative controls. The cells were incubated at 37 °C for 48 hours after which cell-free supernatants were harvested after centrifugation and stored at −80 °C until analysed. Interferon-gamma (IFN-γ) in culture supernatants was measured using ELISA. Briefly, plates (ViewPlate-96, PerkinElmer, Seer Green, UK) were coated with mouse anti-bovine IFN-γ mAb (clone CC330, BioRad Antibodies, Oxford, UK) at 5 μg/ml in 0.05 M carbonate/bicarbonate coating buffer (pH 9.6) (Sigma). Following overnight incubation plates were blocked with 1% bovine serum albumin (BSA) and 0.05% Tween-20. Test supernatant was added to duplicate wells. A two-fold serial dilution of recombinant bovine IFN-γ (BioRad Antibodies) in cRPMI, starting at 50 ng/ml, and medium alone was added as standard/positive and negative controls respectively. The plates were washed and biotin-conjugated mouse anti-bovine IFN-γ mAb at 5 μg/ml (clone CC302, BioRad Antibodies) added. The plates were incubated and washed as above and streptavidin conjugated horseradish peroxidise (SA-HRP; GE Healthcare, Little Chalfont, UK) was added. After a final wash, Supersignal^®^ ELISA Femto Substrate (Thermo Fisher Scientific) was added according to manufacturer’s data sheet, the relative light units (RLU) measured on a luminometer (≈425 nm) (Victor™ × 4 Multiplate Plate Reader; Perkin Elmer, Seer Green, UK).

### Assessment of BVDV viraemia post-challenge

The BVDV envelope RNase glycoprotein, E^rns^, was detected in heparinised blood samples post-challenge using the HerdChek^®^ BVDV Antigen/Serum Plus ELISA kit according to the manufacturer’s instructions (IDEXX Switzerland AG, Liebefeld-Bern, Switzerland). BVDV RNA was extracted from EDTA blood samples post-challenge using the QIAamp Viral RNA Mini Kit (Qiagen, Crawley, UK) as described by the manufacturer. BVDV RNA content measured using a single tube nested quantitative RT-PCR (qRT-PCR) TaqMan^®^ assay as described^69^. Signal was detected using a Stratagene MX3000 P Real-Time PCR machine (Agilent Technologies, La Jolla, CA, USA) and data analysed on the MX Pro 4.1.0c Software (Agilent Technologies). Relative quantities of viral RNA in samples were quantified by interpolation against a 10-fold dilution of RNA extracted from an equivalent volume of virus (BVDV-1a Oregon C24V) of known titre (standard range from 10^7.55^ to 10^0.55^ TCID_50_/ml)^70^.

### Statistical analysis

GraphPad Prism 6.01 (GraphPad Software, La Jolla, USA) was used for graphical and statistical analysis of data sets. A one-way or two-way analysis of variance (ANOVA) was employed to analyse fixed effects on different traits with post-hoc tests as detailed in figure legends. A p value < 0.05 was considered statistically significant.
